# Association of burnout with doctor–patient relationship and common stressors among postgraduate trainees and house officers in Lahore—a cross-sectional study

**DOI:** 10.7717/peerj.5519

**Published:** 2018-09-10

**Authors:** Waqas Ahmad, Huma Ashraf, Afnan Talat, Aleena Ahmad Khan, Ammad Anwar Baig, Iqra Zia, Zohak Sarfraz, Hifsa Sajid, Marium Tahir, Usman Sadiq, Hira Imtiaz

**Affiliations:** CMH Lahore Medical College, Lahore, Pakistan

**Keywords:** Burnout, Distress, Doctor patient relationship, Lahore, Stressors, PPOS, CBI, Patient care, Doctors

## Abstract

**Introduction:**

Burnout is defined as a prolonged state of physical and psychological exhaustion. Doctors, due to the demanding nature of their job, are susceptible to facing burnout, which has far reaching implications on their productivity and motivation. It affects the quality of care they provide to patients, thus eroding the doctor–patient relationship which embodies patient centeredness and autonomy. The study aims at addressing the stressors leading to burnout and its effect on the doctor–patient relationship.

**Methods:**

A descriptive, cross-sectional study design with convenience (non-probability) sampling technique was employed in six major hospitals of Lahore, Pakistan. A total of 600 doctors were approached for the study which included house officers or “HOs” (recent graduates doing their 1 year long internship) and post-graduate trainees or “PGRs” (residents for 4–5 years in their specialties). Burnout was measured using the Copenhagen Burnout Inventor (CBI) while attitudes towards the doctor–patient relationship was measured using the Patient Practitioner Orientation Scale (PPOS), which measures two components of the relationship: power sharing and patient caring. Pearson correlation and linear regression analysis were used to analyze the data via SPSS v.21.

**Results:**

A total of 515 doctors consented to take part in the study (response rate 85.83%). The final sample consisted of 487 doctors. The burnout score was not associated with the total and caring domain scores of PPOS (*P* > 0.05). However, it was associated with the power sharing sub-scale of PPOS. Multiple linear regression analysis yielded a significant model, by virtue of which CBI scores were positively associated with factors such as female gender, feeling of burn out, scoring high on sharing domain of PPOS and a lack of personal control while CBI scores were negatively associated with private medical college education, having a significant other, accommodation away from home and a sense of never ending competition. Burnout levels varied significantly between house officers and post graduate trainees. Twenty-three percent of the participants (mostly house officers) had high/very high burnout levels on the CBI (Kristenson’s burnout scoring). Both groups showed significant differences with respect to working hours, smoking status and income.

**Conclusion:**

Although burnout showed no significant association with total and caring domain scores of PPOS (scale used to assess doctor–patient relationship), it showed a significant association with the power sharing domain of PPOS suggesting some impact on the overall delivery of patient care. Thus, it necessitates the monitoring of stressors in order to provide an atmosphere where patient autonomy can be practiced.

## Introduction

Stress, in moderation, is a driving force for humans to thrive but when present in excess leads, among other things, to burnout—“a state of prolonged physical and psychological exhaustion.” (Dyrbye et al., 2014). Burnout has become an integral part of the lives of medical students (Dyrbye et al., 2014) and practicing health professionals (Dyrbye et al., 2006).

Burnout is a well-documented phenomenon amongst students of medicine worldwide. A study done in Minnesota on medical students found a high (45%) prevalence of burnout. (Dyrbye et al., 2006). Similarly, another study found burnout rates as high as 49.5% among Greek medical students (Msaouel et al., 2010). In 2014, we conducted a study at two medical colleges of Pakistan and discovered that 47% of students had high levels of burnout. In addition, 30% of the students with high levels were not aware of being burnt out (Muzafar et al., 2015). Medical students, due to the uncertainties of the future, rigorous system of evaluation and never-ending need to keep up with the latest trends in the evolving medical field whilst maintaining a social life, inevitably tend to burn out (Dyrbye et al., 2014). It is hypothesised that this will continue to affect the work of students as they progress through medical training as house officers (an MBBS intern working under the supervision of an attending physician) and as postgraduate trainee (resident) doctors.

In a study conducted in Kerala, India, patient related burnout was reported to be the highest among the medical and surgical interns (68.62%) in contrast to non-medical/non-surgical residents. The study also showed a positive correlation between the number of years of residency and the degree of burnout (Ratnakaran, Prabhakaran & Karunakaran, 2016). Moreover, risk factors like working more than 8 hours per day, high workload, and a lack of self-care were linked to burnout (Rizo-Baeza et al., 2018). Both in the work and personal sphere, people expect equality of gain for their investment. A higher level of depersonalization, emotional exhaustion and a decreased sense of personal accomplishment was reported in student nurses who felt that they were committing more to their hospital and patients than what they were receiving back (Schaufeli, Dierendonck & Gorp, 1996). In addition to work, family stressors like being a single parent can also play a role in causing burnout (Rizo-Baeza et al., 2018). In contrast, an empowering leadership leads to more engaged workers and hence causes a decrease in burnout (Laschinger et al., 2015). Healthcare workers with support from their workplace (e.g., from supervisors) reported a decrease in emotional exhaustion, which can ultimately decrease burnout (Constable & Russell, 1986).

Studies have found that many medical trainees experience burnout (Dyrbye et al., 2014) which may be problematic as it might erode a healthy doctor patient relationship; this relationship embodies trust and empathy (Ha & Longnecker, 2010). The hallmark of an accurate diagnosis and the effectiveness of the treatment administered is attributed to the quality of the doctor–patient relationship (Hellin, 2002). A patient-centred or egalitarian relationship gives the patient complete freedom over the choice and approach of his treatment, thus bridging the gap between the doctor and patient with lesser incidence of litigations and complaints (Beckman et al., 1994). On the other hand, a doctor-centred or paternalistic relationship limits the exchange of information and takes away the patient’s control over his treatment. This type of relationship is believed to result from cynicism and the lack of empathy that are linked to burnout (Paro et al., 2014).

A number of studies have been conducted to find the association between burnout and the doctor–patient relationship. A study conducted by Ratanawongsa et al. (2008) showed no association between burnout and differences in the doctor’s behaviour towards their patients. However, burnout did seem to have a positive correlation with the patient’s communication with the physician; patients demonstrated more rapport-building during interactions with physicians who had high burn out (Ratanawongsa et al., 2008). In contrast, a meta-analysis concluded that burnout is attributed to a poor doctor-relationship (Sablik, Samborska-Sablik & Drożdż, 2013). This notion, that burnout causes difficulty in establishing a mutual doctor–patient relationship, still remains questionable.

It is for these reasons and the lack of adequate research and reliable data, particularly from Pakistan (one of the most populated countries), that necessitated us to evaluate the relationship between burnout and the doctor–patient relationship. In lieu of these observations we aim: (1) to assess the level of clinically significant burnout among the sample of post-graduate trainees and house officers; (2) to analyse various personal, professional and organisational factors contributing to a physician’s burnout; (3) to draw a comparison between HOs and PGRs for burnout and its risk factors, and; (4) to explore a possible association of burnout with the doctor–patient relationship and assess attitude of doctors towards the relationship.

## Methodology

### Study sample

A descriptive, cross-sectional study design with convenience (non-probability) sampling technique was employed. In Pakistan after completion of basic medical education (MBBS) doctors embark on 1 year long training post of “House Officer” or “HO” after which they are legally allowed to practice medicine in Pakistan. If a doctor chooses to pursue specialty training they do so by becoming “Post Graduate Residents” or “PGRs” for 4–5 years in their respective specialties. 600 doctors were approached for the study out of which 515 consented to take part (response rate was 85.83%). Twenty-eight respondents were excluded due to lack of basic information e.g., age, gender, etc., along with missing more than three and two responses in PPOS (Patient Practitioner Orientation Scale) and CBI (Copenhagen Burnout Inventory), respectively. The final sample size thus consisted of 487 doctors. Data collection was done entirely in Lahore city among various hospitals, both public and private, between August 2017 and December 2017. A series of demographic questions were included in the questionnaire to record some basic information.. A comparison between the postgraduate trainees and house officers was made in this research.

### Instruments

#### Copenhagen Burnout Inventory

Copenhagen Burnout Inventory (CBI) was employed which measures three aspects of burnout; Personal, Work related and Patient related. Only personal sub-domain of burnout was assessed in this study with a six-item scale. Cronbach’s alpha is 0.80. All burnout items were intermixed. The responses have the following categories: Always, Often, Sometimes, Seldom, Never/almost never. Each corresponds to a score of 100, 75, 50, 25, and 0. The total score on the scale is the average of the scores on the items. Kristensen’s classification was also used which divides the scores by setting up cut off points such as: nil, low, moderate, high and very high burnout, where: nil=score equal to lowest 25% of distribution of scores, low=score of next higher 25% of distribution of scores, moderate=score of the next 12.5% of distribution of scores, high=score of the next 12.5% of distribution of scores, and very high=score of the highest 25% of distribution of scores (Winwood & Winefield, 2004; Kristensen et al., 2005).

#### Patient Practitioner Orientation Scale

The attitudes of participants towards doctor–patient relationship were assessed by using a reliable instrument called Patient Practitioner Orientation Scale (PPOS) (Krupat et al., 2000). The PPOS contains 18-items and measures the subject’s leaning towards a doctor-centred or a patient-centred care. Cronbach’s alpha is 0.83. Each item has six possible responses ranging from 1 (strongly agree) to 6 (strongly disagree). PPOS also contains two subscales which measure two sub-domains of doctor–patient relationship: Sharing and Caring. Sharing refers to an individual’s belief that a patient should have the right to be an equal part of decision making process along with the physician. Caring refers to a doctor’s belief that a patient should be treated as a whole rather than just as a disease. Both sub-scales have nine items each. All the scores are reported as mean of the total score ranging from 1 (doctor centred) to 6 (patient centred).

#### Stressors’ scale

Stressful events were measured on a 30 item questionnaire which has a five point likert scale ranging from none of the time to all of the time. The headings are classified as: lifestyle, psychological, work and personal stressors.

### Statistical analysis

SPSS Inc., (Chicago, IL, USA) version 21 software was used for analysis. Descriptive statistics are shown for age, gender, level of training, etc. (Table 1). Independent-sample *t*-test was used for the comparison of means. Five groups of responses for stressful events (None of the time, Rarely, Some of the time, Often and All of the time) were regrouped into two groups for the simplicity of analysis. None of the time, rarely and some of the time were grouped together, while often and all of the time were grouped together. Multiple Linear Regression analysis was run to analyse association of scores on CBI (dependent variable) with demographic characteristics of respondents, age, income, stressors and PPOS as predictor variables. Histogram and P-P plots were visualized to assess the assumption of normality of data. Durbin Watson test, Cook’s distance, case wise diagnostics and values of variance inflation factor (VIF), tolerance (TOL) were run to ensure all assumptions of multiple regression analysis were met. Categorical variables were re-coded as dummy variables with values 0 and 1. The variables with more than 1 option e.g., relationship status were regrouped into two logical groups (having a significant other and having no significant other) for a robust regression model (final sample, *N* = 487).

**Table 1:** Demographics of the participants (*N* = 487).

**Parameter**	*n* (%)
**Mean age ± SD**	26.39 ± 2.97
**Gender**	
Male	216(44.4)
Female	271(55.6)
**Level of training**	
HO	282(58.0)
PGR	205(42.0)
**Living status**	
With family	243(49.9)
Hostel	244(50.1)
**Relationship status**	
Single	289(59.3)
Married	114(23.4)
In a relationship	27(5.5)
Engaged	56(11.5)
Divorced	1(0.2)

#### Ethical statement

Ethical approval (reference number 40 ERC/CMHLMC) from Ethics Review Committee of CMH Lahore Medical College and Institute of Dentistry was obtained. Informed written consent was sought from each participant and their personal details were kept confidential.

## Results

### Demographics

Demographic characteristics of the participants are shown in Table 1. According to Table 1, the sample at hand is quite young and represents both genders almost equally. More HOs than PGRs took part in the study, which simply reflects their abundance over PGRs.

### Risk factors

Mean PPOS score of the entire sample was 3.63 ± 0.45. Subscale scores for caring and sharing domain were 3.92 ± 0.55 and 3.34 ± 0.67 respectively. CBI and total PPOS score along with caring domain had no significant linear correlation. Sharing domain, however, was significantly correlated with CBI scores in the multiple linear regression model (Table 2). Total PPOS, sharing and caring domain yielded no significant multiple regression models with demographics and stressful events.

**Table 2:** Significant determinants of burnout (regression analysis) (*N* = 487).

Model	Unstandardized coefficients		*p*-value	95.0% Confidence interval for B	
	B	Std. error		Lower bound	Upper bound
(Constant)	31.298	12.050	.010	7.616	54.981
Participant’s Gender (female)	6.035	1.815	<0.001	2.468	9.602
Graduated from a Private Institute	−7.335	2.438	.003	−12.125	−2.544
Relationship status?	−3.420	1.679	.042	−6.721	−.120
Burn-out is a state of prolonged physical and psychological exhaustion. Do you feel burnt out?	11.794	1.709	.001	8.435	15.153
SharingPPOS (mean)	2.510	1.235	.043	.083	4.938
CaringPPOS (mean)	−1.407	1.465	.338	−4.287	1.474
Accommodation away from home.	−5.007	1.938	.010	−8.815	−1.198
Lack of personal control over what you do.	5.160	2.280	.024	.679	9.642
Sense of never ending competition.	−6.896	1.828	<0.001	−10.488	−3.304

**Notes.**

*R* = 0.637, *R*_2_ = 0.405, *R*_3_ = 0.405, Durbin Watson = 1.846, Dependent Variable: CBI Score.

Mean CBI scores were 51.18 ± 20.29. CBI scores yielded a significant regression model with the demographics and stressful events (Table 2). High scores on CBI were positively associated with female gender, perception of being burnt out, scoring high on sharing domain of PPOS and lack of personal control over what one does. Similarly graduating from a private medical college (Table 2), having a significant other (Table 2), accommodation away from home (Table 2) and sense of never ending competition (Table 2) were negatively correlated with CBI scores. All other predicator variables did not contribute significantly towards predicting CBI scores. Of the participants stating self-perception of burnout, only 36% had high/very high burnout levels.

### Comparison

A comparison between HO and PGRs was made in Table 3 where they are compared by Pearson chi-square and Independent Sample *t*-test.

**Table 3:** A comparison of house officers and post graduate residents.

Variable (total *n* = 487)	House officer (*n* = 282) *n*(%)	Post graduate resident (*n* = 205) *n*(%)	*p*-value
No income or ≤ USD 312/month^b^	93.6	20.5	<0.001
Income ≥ USD 312/Month^b^	6.4	79.5	<0.001
Graduated from govt. institute^b^	62.4	81.5	<0.001
Graduated from private institute^b^	37.6	18.5	<0.001
Smoke^b^	7.8	17.1	<0.001
Burn-out is a state of prolonged physical and psychological exhaustion. Do you feel burnt-out?^b^	62.4	45.4	<0.001
High/Very High Burnout (According to Kristensen’s Classification of CBI)^b^	29.8	16.1	<0.001
Wouldn’t change their profession if had the option^b^	65.2	56.6	0.033
Working hours/week mean (±SD)^a^	65.09 ± 16.01	58.15 ± 14.69	<0.001

**Notes.**

a Independent sample *t*-test.

b Pearson Chi-Square.

## Discussion

In our study, the first hypothesis that house officers (HOs) and post graduate trainees (PGRs) would have a high burnout rate was supported by 29.8% of HOs and 16.1% of PGRs reporting high/very high burnout. A number of studies reported similar results (Al-Dubai et al., 2013; Shanafelt et al., 2010; Ratanawongsa et al., 2008; Shanafelt et al., 2015; Guthrie et al., 1998; Dyrbye et al., 2010; Marjani et al., 2008; Dyrbye et al., 2014; Ratnakaran, Prabhakaran & Karunakaran, 2016).

This observation can be explained by the emotionally depleting nature of the job, long working hours (Khamisa, Peltzer & Oldenburg, 2013), patient overload (Khamisa, Peltzer & Oldenburg, 2013) and challenging work environment. These factors may account for the significant difference observed between burnout scores of public and private hospitals (Table 2, *p* = 0.003). Moreover, our study showed that a high percentage of doctors with burnout reported feeling burnt out, showing self-awareness of their state. (Table 2, *p* < 0.001). Self-awareness is essential in order to seek help (Rickwood et al., 2005). This may prove to have a role in prevention of burnout. It was also found that females have a higher tendency for being burnt out as compared to males (Table 2, *p* < 0.001).

Another interesting finding was the negative relationship between the sense of never ending competition and burnout (Table 2, *p* < 0.001). Medical schools are known for their low acceptance rates and high academic merit. In addition to these, the type A personality of medical students and a continuous need for academic excellence can make medicine competitive. This finding indicates a positive attitude of trainees towards competition which is considered to be conducive for learning. Friendly competition has been linked to stronger motivation and improved performance (Burguillo, 2010); motivation is a key component in preventing burnout (Al-Dubai et al., 2013).

Moreover, social support which was reported as an important preventive factor of burnout (Chou, Li & Hu, 2014), was supported by our study, which found reduced levels of burnout among doctors having a significant other (Table 2, *p* = 0.042). In contrast, accommodation away from home was reported to have a negative association with burnout (Table 2, *p* = 0.01). This can be explained with improved communication, better transport and hostel facilities which may compensate for homesickness. Accommodation at hostels increases social interaction and support offered by peers experiencing similar problems, which may help in reducing burnout. Previously, lack of peer support has been linked with increased levels of burnout (Khamisa, Peltzer & Oldenburg, 2013).

During the study a comparison was drawn between burnout rates of HOs and PGRs. It was observed that more HOs had a “high/ very high burnout” than PGRs (Table 3, *p* < 0.001). This was comparable to an Indian study which found high rates of personal burnout among HOs than PGRs (Ratnakaran, Prabhakaran & Karunakaran, 2016). The high level of burnout can be attributed to one major common factor: stressful nature of early medical training. HOs work longer hours with lower income than PGRs (Table 3, *p* < 0.001). Greater number of working hours lead to poor work-life balance leaving less personal time (Shanafelt et al., 2015) and increases patient contact which causes emotional strain for the doctor (Sablik, Samborska-Sablik & Drożdż, 2013). As time for recreation is important for regeneration of emotional energy, working hours need to be addressed with a decrease in number of hours to 48 hours as suggested by European Working Time Directive (EWTD). Low income implies more financial strain, an important stressor that affects quality of life, and lack of reward. The resulting fatigue caused by it may also increase stress.

However, HOs were found to be more self-aware, as they had a higher rate of self-reported feeling of burnout than PGRs and still opted not to change their profession showing more resilience. Healthy coping mechanisms are linked to resilience (Hamill, 2003) which may be the reason for lower percentage of HOs (Table 3, *p* < 0.001) smoking (7.8%) compared to PGRs trainees (17.1%). Smoking, an unhealthy strategy, is a popular choice to cope with stress among Pakistani medical students(Shaikh et al., 2004).

Doctors showed a tendency towards doctor centered relationship. This is comparable to a previous research where MBBS students showed a similar attitude towards doctor–patient relationship (Ahmad et al., 2015). There is lack of educational interventions like communication skills’ trainings aimed at improving doctor–patient relationship, which may be the reason for similarity of the attitude. Although burnout showed no significant association with total score and caring domain of PPOS, it showed significant association with power sharing domain of PPOS (Table 2, *p* = 0.083). Sharing implies equal autonomy of the patient in making decisions, which requires time and effort on part of the physician to patients’ concerns and their choices, resulting in exhaustion and inefficacy. Similarly, lack of control over what one does also showed to have a significant association with high burnout (Table 2, *p* = 0.024) in this study and a previous study (Richter et al., 2014). Given that sense of autonomy and efficacy are important determinants of intrinsic motivation (Ryan & Deci, 2000), it can be inferred that more power sharing results in lack of intrinsic motivation thus leading to burnout. Another study indicated doctor–patient interaction as an important stressor of burnout (Sablik, Samborska-Sablik & Drożdż, 2013). It explained that the helpers have to over-extend themselves to the point where they become emotionally saturated which onsets emotional exhaustion. When feeling exhausted, it is difficult to gain a sense of accomplishment, resulting in inefficacy (Bresó, Salanova & Schaufeli, 2007) that eventually leads to burnout.

## Conclusion

Despite high rates of burnout and its impact on patient care, little research and preventive measures have been taken against it. Thus, it is necessary to comprehend as well as establish the causal relation between burnout and doctor patient relationship. Moreover, to improve care delivery, it is essential to regulate the stressors that decrease the efficacy of doctors as exhibited by this study such as; long working hours, low income, emotional depletion, lack of autonomy with an evident preponderance of organizational over personal factors. However, it does not imply association of burnout with doctor–patient relationship, it only signifies relation to power sharing domain of doctor–patient relationship.

## Limitations

(1) A cross-sectional study design rather than a case-control was used to study association of burnout and doctor–patient relationship. (2) Self-assessment questionnaires were employed to study burnout and doctor–patient relationship. It would have been more reliable to study behaviour of doctors or assess doctor–patient relationship through patient feedback. (3) It is suggested for future research to study other factors that may have an impact on doctor–patient relationship such an examination intervention (Hur et al., 2014). (4) A convenience sampling technique was employed but it is suggested for future research to use a random sampling technique.

## Supplemental Information

10.7717/peerj.5519/supp-1 Supplemental Information 1 Questionnaire

10.7717/peerj.5519/supp-2 Data S1 Raw data

10.7717/peerj.5519/supp-3 Table S1 Raw results including insignificant and significant determinants of burnout

## Additional Information and Declarations

### Data Availability

The following information was supplied regarding data availability: Raw data inludes: “Raw data.spss” which has forms that were coded and entered in SPSS.v5)and “Table 2 Raw result (regression analysis)” which has both significant and insignificant determinants of burnout The raw data are provided in the Supplemental Files.
