# Supplementary material for: Association of burnout with doctor–patient relationship and common stressors among postgraduate trainees and house officers in Lahore—a cross-sectional study
Source: PeerJ. 2018 Sep 10;6:e5519. doi: 10.7717/peerj.5519 (PMC6136394; doi:10.7717/peerj.5519)
Supplement: Supplemental Information 1 [file peerj-06-5519-s001.doc]

**Burnout and Doctor-Patient Relationship: A cross-sectional study on HOs and PGRs of Pakistan.**

**Age: _____** Years. **Graduated from:** Govt. / Private Institute

**Gender:** M F  **Working in:** Govt. / Private Hospital

**Level of training: HO PGR**

***(For HOs only)* *(For PGRs Only)***

It’s been ________ months since my house-job started. Specialty of training: ______________________________.

Year of training: 1^st^ /2^nd^  /3^rd^  /4^th^ /5^th^

*(Read each statement and choose the option which best describes you)*

| **Sr** | **Statement** | **Always** | **Often** | **Sometimes** | **Seldom** | **Never/almost never** |
| --- | --- | --- | --- | --- | --- | --- |
| **1** | How often do you feel tired? | * | * | * | * | * |
| **2** | How often are you physically exhausted? | * | * | * | * | * |
| **3** | How often are you emotionally exhausted? | * | * | * | * | * |
| **4** | How often do you think: “I can’t take it anymore”? | * | * | * | * | * |
| **5** | How often do you feel worn out? | * | * | * | * | * |
| **6** | How often do you feel weak and susceptible to illness? | * | * | * | * | * |

**Q.1: Do you live with your?**

a) family.

b) in hostel away from family.

**Q.2: Relationship status:** a) Single b) Married c) In a relationship d) Engaged e) Divorced

**Q.3: How many hours, on average, you work in a week?**

_________ Hours/Week.

**Q.4: Your monthly income falls in which category (Per Month)?**

a) I work at an honorary post.

b) Low Income (≤ Rs. 8, 500)

c) Lower-Middle Income (≥ Rs 8, 501 and ≤ Rs. 33, 000)

d) Upper-Middle Income (≥ Rs. 33, 001 and ≤ Rs.102, 000)

e) High-Income (> Rs.102, 001)

**Q.5: Do you smoke?**

a) No. b) Yes. (Specify the number: ________ /Day)

**Q.6: Do you do exercise as a part of your daily routine (***150 mins/week of moderate or 75 min/week of vigorous activity***)?**

a) No. b) Yes.

**Q.7: Burn-out is a state of prolonged physical and psychological exhaustion. Do you feel burnt-out?**

a) No. b) Yes.

­

**Q.8: If you could go back in time, would you choose a different profession?**

a) Yes b) No.

*The statements below refer to beliefs that people might have concerning doctors, patients, and medical care. Read each item and then encircle in the circle to indicate how much you agree or disagree with each.*

| **Sr** | **Statement** | **Strongly disagree** | **Moderately Disagree** | **Slightly Disagree** | **Slightly Agree** | **Moderately Agree** | **Strongly Agree** |
| --- | --- | --- | --- | --- | --- | --- | --- |
| **1** | The doctor is the one who should decide what gets talked about during a visit. | ***** | ***** | ***** | ***** | ***** | ***** |
| **2** | Although health care is less personal these days, this is a small price to pay for medical advances. | ***** | ***** | ***** | ***** | ***** | ***** |
| **3** | The most important part of the standard medical visit is the physical exam. | ***** | ***** | ***** | ***** | ***** | ***** |
| **4** | It is often best for patients if they do not have a full explanation of their medical condition. | ***** | ***** | ***** | ***** | ***** | ***** |
| **5** | Patients should rely on their doctors’ knowledge and not try to find out about their conditions on their own | ***** | ***** | ***** | ***** | ***** | ***** |
| **6** | When doctors ask a lot of questions about a patient’s background, they are prying too much into personal matters. | ***** | ***** | ***** | ***** | ***** | ***** |
| **7** | If doctors are truly good at diagnosis and treatment, the way they relate to patients is not that important. | ***** | ***** | ***** | ***** | ***** | ***** |
| **8** | Many patients continue asking questions even though they are not learning anything new. | ***** | ***** | ***** | ***** | ***** | ***** |
| **9** | Patients should be treated as if they were partners with the doctor, equal in power and status. | ***** | ***** | ***** | ***** | ***** | ***** |
| **10** | Patients generally want reassurance rather than information about their health. | ***** | ***** | ***** | ***** | ***** | ***** |
| **11** | If a doctor’s primary tools are being open and warm, the doctor will not have a lot of success. | ***** | ***** | ***** | ***** | ***** | ***** |
| **12** | When patients disagree with their doctor, this is a sign that the doctor does not have the patient’s respect and trust. | ***** | ***** | ***** | ***** | ***** | ***** |
| **13** | A treatment plan cannot succeed if it is in conflict with a patient’s lifestyle or values. | ***** | ***** | ***** | ***** | ***** | ***** |
| **14** | Most patients want to get in and out of the doctor’s office as quickly as possible. | ***** | ***** | ***** | ***** | ***** | ***** |
| **15** | The patient must always be aware that the doctor is in charge. | ***** | ***** | ***** | ***** | ***** | ***** |
| **16** | It is not that important to know a patient’s culture and background in order to treat the person’s illness. | ***** | ***** | ***** | ***** | ***** | ***** |
| **17** | Humor is a major ingredient in the doctor’s treatment of the patient. | ***** | ***** | ***** | ***** | ***** | ***** |
| **18** | When patients look up medical information on their own, this usually confuses more than it helps. | ***** | ***** | ***** | ***** | ***** | ***** |

(Out of the following specify the frequency of each stressful event you experience in your daily life)

| **Sr** | **Event** | **None of the time** | **Rarely** | **Some of the time** | **Often** | **All of the time** |
| --- | --- | --- | --- | --- | --- | --- |
| **LIFESTYLE** | | | | | | |
| **1** | Too Much Work With Little Balance | 1 | 2 | 3 | 4 | 5 |
| **2** | No Help or Supportive Resources | 1 | 2 | 3 | 4 | 5 |
| **3** | Too Little Social Support | 1 | 2 | 3 | 4 | 5 |
| **4** | Lack of time for recreation. | 1 | 2 | 3 | 4 | 5 |
| **5** | Too Little Sleep | 1 | 2 | 3 | 4 | 5 |
| **6** | Too Little Time Off | 1 | 2 | 3 | 4 | 5 |
| **7** | Lack of close, supportive relationships | 1 | 2 | 3 | 4 | 5 |
| **8** | Accommodation away from home | 1 | 2 | 3 | 4 | 5 |
| **PSYCHOLOGICAL** | | | | | | |
| **9** | Lack of Belief in What You Do | 1 | 2 | 3 | 4 | 5 |
| **10** | Choosing field of medicine, against your interest, on family pressure. | 1 | 2 | 3 | 4 | 5 |
| **Work** | | | | | | |
| **11** | High-Stress Times with No “Down” Times | 1 | 2 | 3 | 4 | 5 |
| **12** | Big Consequences for Failure | 1 | 2 | 3 | 4 | 5 |
| **13** | Lack of Personal Control over what you do | 1 | 2 | 3 | 4 | 5 |
| **14** | Insufficient rewards or acknowledgement of your work. | 1 | 2 | 3 | 4 | 5 |
| **15** | Poor Communication with colleagues and seniors | 1 | 2 | 3 | 4 | 5 |
| **16** | Poor Leadership skills of training supervisors | 1 | 2 | 3 | 4 | 5 |
| **17** | Doing work that’s monotonous or unchallenging | 1 | 2 | 3 | 4 | 5 |
| **18** | Long working hours | 1 | 2 | 3 | 4 | 5 |
| **19** | Patient overload. | 1 | 2 | 3 | 4 | 5 |
| **20** | Illegitimate *political*, *administrative* etc. pressure. | 1 | 2 | 3 | 4 | 5 |
| **21** | Discrimination at work by colleagues due to your gender. | 1 | 2 | 3 | 4 | 5 |
| **22** | Discrimination at work by colleagues due to your role in society (e.g. being a wife and guardian of the household) | 1 | 2 | 3 | 4 | 5 |
| **23** | Discrimination at work by colleagues due to your caste. | 1 | 2 | 3 | 4 | 5 |
| **24** | Sense of never ending competition. | 1 | 2 | 3 | 4 | 5 |
| **PERSONAL** | | | | | | |
| **25** | Family responsibilities. | 1 | 2 | 3 | 4 | 5 |
| **26** | Uncertain future and limited opportunities to prosper. | 1 | 2 | 3 | 4 | 5 |
| **27** | Loss of a family member in past 12 months. | 1 | 2 | 3 | 4 | 5 |
| **28** | Witnessed a major illness of a closed-family member. | 1 | 2 | 3 | 4 | 5 |
| **29** | High parental Expectations | 1 | 2 | 3 | 4 | 5 |
| **30** | Financial strain | 1 | 2 | 3 | 4 | 5 |
