# Supplementary material for: Association of burnout with doctor–patient relationship and common stressors among postgraduate trainees and house officers in Lahore—a cross-sectional study
Source: PeerJ. 2018 Sep 10;6:e5519. doi: 10.7717/peerj.5519 (PMC6136394; doi:10.7717/peerj.5519)
Supplement: Table S1 [file peerj-06-5519-s003.docx]

|  | | | | | | | | | |
| --- | --- | --- | --- | --- | --- | --- | --- | --- | --- |
| Model | | Unstandardized Coefficients | | Standardized Coefficients | t | Sig. | 95.0% Confidence Interval for B | |  |
|  |  | B | Std. Error | Beta |  |  | Lower Bound | Upper Bound |  |
|  | (Constant) | 31.298 | 12.050 |  | 2.597 | .010 | 7.616 | 54.981 |  |
|  | **Participant's Gender (Female)** | **6.035** | **1.815** | **.148** | **3.326** | **.001** | **2.468** | **9.602** |  |
|  | **Graduated from a Private Institute (YES)** | **-7.335** | **2.438** | **-.165** | **-3.009** | **.003** | **-12.125** | **-2.544** |  |
|  | **Relationship Status? (Significant other present)** | **-3.420** | **1.679** | **-.083** | **-2.037** | **.042** | **-6.721** | **-.120** |  |
|  | **Burn-out is a state of prolonged physical and psychological exhaustion. Do you feel burnt-out? (YES)** | **11.794** | **1.709** | **.289** | **6.900** | **.000** | **8.435** | **15.153** |  |
|  | **SharingPPOS_mean** | **2.510** | **1.235** | **.083** | **2.032** | **.043** | **.083** | **4.938** |  |
|  | CaringPPOS_mean | -1.407 | 1.465 | -.039 | -.960 | .338 | -4.287 | 1.474 |  |
|  | **Accommodation away from home. (Yes)** | **-5.007** | **1.938** | **-.123** | **-2.584** | **.010** | **-8.815** | **-1.198** |  |
|  | **Lack of personal control over what you do. (Yes)** | **5.160** | **2.280** | **.108** | **2.263** | **.024** | **.679** | **9.642** |  |
|  | **Sense of never ending competition. (Yes)** | **-6.896** | **1.828** | **-.169** | **-3.773** | **.000** | **-10.488** | **-3.304** |  |
|  |  |  |  |  |  |  |  |  |  |
| a. Dependent Variable: CBI_ScoreMean | | | | | | | | | |

| **Model Summary^b^** | | | | | | | | | | |
| --- | --- | --- | --- | --- | --- | --- | --- | --- | --- | --- |
| Model | R | R Square | Adjusted R Square | Std. Error of the Estimate | Change Statistics | | | | | Durbin-Watson |
|  |  |  |  |  | R Square Change | F Change | df1 | df2 | Sig. F Change |  |
| 1 | .637^a^ | .405 | .345 | 16.42385 | .405 | 6.684 | 45 | 441 | .000 | 1.846 |
| a. Predictors: (Constant), Financial strain., If you could go back in time, would you choose a different profession?, Working at Govt. or Private Hospital, SharingPPOS_mean, Relationship Status?, CaringPPOS_mean, How many hours, on average, you work in a week?, Illegitimate political, administrative etc. pressure., Do you smoke?, Choosing field of medicine, against you interest, on family pressure., Witnessed a major illness of closed-family member., Do you exercise as a part of your daily routine?, Burn-out is a state of prolonged physical and psychological exhaustion. Do you feel burnt-out?, Poor leadership skills of training supervisors., Discrimination at work by colleagues due to your caste., Accommodation away from home., Your monthly income falls in which category (Per month)?, Lack of time for recreation., Big consequences for failure., High parental expectations., Lack of Belief in what you do ., Long working hours., Lack of close, supportive relationships., Loss of family member in past 12 months., Discrimination at work by colleagues due to your gender., Too little social support., Participant's Gender, Doing work that's monotonous or unchallenging., Sense of never ending competition., Family responsibilitis., Insufficient rewards or acknowledgement of your work., Uncertain furture and limited opportunities to prosper., Patient overload., Participant's Age, Lack of personal control over what you do., No help or supportive resources., High stress times with no "Down" time., Do you live with your?, Too little sleep., Discrimination at work by colleagues due to your role in society (e.g. being a wife and guardian of the household)., Too much work with little balance., Too little time off., Poor communication with colleagues and seniors., Graduated from Govt. or Private Institute, Level of Training | | | | | | | | | | |
| b. Dependent Variable: CBI_ScoreMean | | | | | | | | | | |

| **ANOVA^a^** | | | | | | |
| --- | --- | --- | --- | --- | --- | --- |
| Model | | Sum of Squares | df | Mean Square | F | Sig. |
| 1 | Regression | 81135.887 | 45 | 1803.020 | 6.684 | .000^b^ |
|  | Residual | 118956.587 | 441 | 269.743 |  |  |
|  | Total | 200092.474 | 486 |  |  |  |
| a. Dependent Variable: CBI_ScoreMean | | | | | | |
| b. Predictors: (Constant), Financial strain., If you could go back in time, would you choose a different profession?, Working at Govt. or Private Hospital, SharingPPOS_mean, Relationship Status?, CaringPPOS_mean, How many hours, on average, you work in a week?, Illegitimate political, administrative etc. pressure., Do you smoke?, Choosing field of medicine, against you interest, on family pressure., Witnessed a major illness of closed-family member., Do you exercise as a part of your daily routine?, Burn-out is a state of prolonged physical and psychological exhaustion. Do you feel burnt-out?, Poor leadership skills of training supervisors., Discrimination at work by colleagues due to your caste., Accommodation away from home., Your monthly income falls in which category (Per month)?, Lack of time for recreation., Big consequences for failure., High parental expectations., Lack of Belief in what you do ., Long working hours., Lack of close, supportive relationships., Loss of family member in past 12 months., Discrimination at work by colleagues due to your gender., Too little social support., Participant's Gender, Doing work that's monotonous or unchallenging., Sense of never ending competition., Family responsibilitis., Insufficient rewards or acknowledgement of your work., Uncertain furture and limited opportunities to prosper., Patient overload., Participant's Age, Lack of personal control over what you do., No help or supportive resources., High stress times with no "Down" time., Do you live with your?, Too little sleep., Discrimination at work by colleagues due to your role in society (e.g. being a wife and guardian of the household)., Too much work with little balance., Too little time off., Poor communication with colleagues and seniors., Graduated from Govt. or Private Institute, Level of Training | | | | | | |

| **Residuals Statistics^a^** | | | | | |
| --- | --- | --- | --- | --- | --- |
|  | Minimum | Maximum | Mean | Std. Deviation | N |
| Predicted Value | 12.1187 | 91.9680 | 51.1893 | 12.92077 | 487 |
| Std. Predicted Value | -3.024 | 3.156 | .000 | 1.000 | 487 |
| Standard Error of Predicted Value | 2.613 | 9.603 | 4.981 | .820 | 487 |
| Adjusted Predicted Value | 11.7228 | 91.1218 | 51.2185 | 13.02738 | 487 |
| Residual | -48.60859 | 54.25115 | .00000 | 15.64502 | 487 |
| Std. Residual | -2.960 | 3.303 | .000 | .953 | 487 |
| Stud. Residual | -3.210 | 3.515 | -.001 | 1.004 | 487 |
| Deleted Residual | -57.17937 | 61.42986 | -.02923 | 17.38076 | 487 |
| Stud. Deleted Residual | -3.244 | 3.561 | -.001 | 1.006 | 487 |
| Mahal. Distance | 11.307 | 165.150 | 44.908 | 15.155 | 487 |
| Cook's Distance | .000 | .039 | .002 | .004 | 487 |
| Centered Leverage Value | .023 | .340 | .092 | .031 | 487 |
| a. Dependent Variable: CBI_ScoreMean | | | | | |
